# Supplementary material for: Impact of the Universal Implementation of Adolescent Hepatitis B Vaccination in Spain
Source: Vaccines (Basel). 2024 May 1;12(5):488. doi: 10.3390/vaccines12050488 (PMC11125626; doi:10.3390/vaccines12050488)
Supplement: Supplementary file 1 [file vaccines-12-00488-s001.zip › vaccines-2952853-SI.pdf]

Supplementary Table 1. Ages in 2005-2021 of persons who were 12 years old in 1991-1996.

	2005	2006	2007	2008	2009	2010	2011	2012	2013	2014	2015	2016	2017	2018	2019	2020	2021
**1991**	26	27	28	29	30	31	32	33	34	35	36	37	38	39	40	41	42
**1992**	25	26	27	28	29	30	31	32	33	34	35	36	37	38	39	40	41
**1993**	24	25	26	27	28	29	30	31	32	33	34	35	36	37	38	39	40
**1994**	23	24	25	26	27	28	29	30	31	32	33	34	35	36	37	38	39
**1995**	22	23	24	25	26	27	28	29	30	31	32	33	34	35	36	37	38
**1996**	21	22	23	24	25	26	27	28	29	30	31	32	33	34	35	36	37

Note: 12 years was the average age at which vaccination began.
